# Antidiabetic potentials of crude and purified sulphated polysaccharides isolated from *Gracilaria gracilis*, a seaweed from South Africa

**DOI:** 10.1016/j.heliyon.2024.e35729

**Published:** 2024-08-02

**Authors:** Leah R. Pillay, Tosin A. Olasehinde, Kolawole A. Olofinsan, Ochuko L. Erukainure, Md. Shahidul Islam, Ademola O. Olaniran

**Affiliations:** a Department of Microbiology, School of Life Sciences, University of KwaZulu-Natal, Westville Campus, Durban, 4000, South Africa; b Department of Biochemistry, School of Life Sciences, University of Kwazulu-Natal, Westville Campus, Durban, 4000, South Africa; c Department of Pharmacology, Faculty of Health Sciences, University of the Free State, Bloemfontein, 9300, South Africa; d Nutrition and Toxicology Division, Food Technology Department, Federal Institute of Industrial Research Oshodi, Nigeria

**Keywords:** Antidiabetic, *Gracilaria gracilis*, Red seaweed, South Africa, Sulphated polysaccharides

## Abstract

Over 90 % of all cases of diabetes that have been diagnosed are type 2 diabetes (T2D), a disease exacerbated by an increase in sedentary behaviour, bad eating habits, and obesity. This study investigated the antidiabetic properties of *Gracilaria gracilis*, using *in vitro* and *ex vivo* experimental models. The sulphated polysaccharides (SPs) from crude extracts of the seaweed powder was prepared via hot (100°C) and cold (25°C) aqueous extraction procedures before purification via an anion exchange chromatographic technique. Both the crude and purified extracts were characterised by Fourier-transform infrared spectroscopy (FT-IR), LC-MS analysis, and Nuclear Magnetic Resonance (NMR) spectroscopy. The crude cold-aqueous and purified hot-aqueous SPs from *G. gracilis* had the strongest α-glucosidase inhibitory effect with IC_50_ value of 0.15 and 0.07 mg/ml, respectively. The purified cold-aqueous SP was the most potent inhibitor of α-glucosidase with an IC_50_ value of 0.17 mg/ml. The crude and purified SP-rich extracts inhibited pancreatic lipase (hot aqueous SP = 0.03 mg/ml) activity and effectively stimulated glucose uptake in yeast cells. Moreover, they showed significantly (*p* < 0.05) better intestinal glucose absorption inhibitory properties at the highest concentration (1 mg/ml) and displayed significantly (*p* < 0.05) better muscle glucose uptake compared to the commercial antidiabetic drug, metformin, at the same concentration. Overall, the current findings indicate that *G. gracilis* SPs may inhibit carbohydrate-hydrolysing enzymes, limit the release of simple sugars from the gut whilst effectively stimulating the use of glucose by peripheral tissue thus may be suitable to develop antidiabetic food supplements after further animal and clinical trials.

## Highlights

• Sulphated polysaccharides were isolated from *G. gracilis*.
• Crude and purified sulphated polysaccharides from *G. gracilis* inhibited carbohydrates and lipids digestive enzymes.
• Crude and purified sulphated polysaccharides from *G. gracilis* inhibited intestinal glucose absorption.
• Crude and purified sulphated polysaccharides from *G. gracilis* stimulated muscle glucose uptake.

## 1 Introduction

Type 2 diabetes mellitus (T2DM) is characterized by a gradual reduction of insulin production from the pancreatic β-cell because of β-cell dysfunction and insulin resistance in the target organs which ultimately turns to chronic hyperglycaemia [1]. Severely high blood glucose levels can cause cardiovascular problems, nephropathy, neuropathy, and retinopathy [2]. There are currently 537 million individuals (20–79 years old) who have diabetes. The International Diabetes Federation (IDF) has predicted that there will be 643 million people living with diabetes by the year of 2030 [3]. According to IDF, 6.7 million deaths in 2021 were due to diabetes mellitus (DM); this translates to 1 fatality from DM in every 5 s. Over and above, impaired glucose tolerance has been identified in 541 million people, which puts an additional burden to increase the risk of developing T2DM [3].

Since T2DM is associated with multiple factors, understanding its complexity is important for the prevention and treatment [4]. The antidiabetic drugs presently used in the pharmaceutical industry play an important role in managing T2DM. However, none of them are without side effects including diarrhoea, bloating, flatulence, and abdominal discomfort [5]. Because of this, it is important to develop novel antidiabetic drugs, with better potencies and minimal or no side effects, when seaweeds are considered as one of the potential sources for many reasons.

In the past decade, seaweeds have gained substantial status in terms of their usefulness as medicinal plant species [6,7]. The consumption of seaweeds as popular cuisines in numerous East-Asian communities has been linked to decreased incidence of cardiovascular disease, hyperlipidaemia and even breast cancer [8]. Unsurprisingly in this region, dietary alteration to Westernised cuisine with reduced daily intake of locally consumed seaweeds have led to higher incidence of lifestyle pathologies such as diabetes [9]. Thus, there is a need to understand how seaweed could elicit these therapeutic benefits.

*Gracilaria* sp. is the third largest genus in the red seaweed group (Rhodophyta) with over 150 species documented globally [10]. While the seaweed species are used in soups and salad preparation in countries like China, Korea, Thailand, Japan and recently in the United States, and they are also utilised for producing about 50% of agar media supplied worldwide [11]. However, in South Africa, *Gracilaria* sp. is used as feed for farmed Abalone, an edible sea snail [12]. According to previous studies, *Gracilaria* sp. is a rich source of bioactive compounds such as sulphated polysaccharides (SPs) that have been identified as potential antidiabetic agents [13]. Moreover, SPs such as sulphated galactans derived from red seaweeds have been reported to exhibit anti-viral, anticoagulant, and anti-inflammatory activities [8,14]. In a previous study, Makkar and Chakraborty [15] reported that *Gracilaria opuntia* is a potential source of bioactive sulphated polygalactans to use as a functional food supplement to deter inflammation and T2DM.

Although there are studies on SPs derived from other red seaweeds [15], there is a scarcity of information on the bioactivity of similar sulphur-containing carbohydrate complexes in *Gracilaria gracilis.* In this study, the antidiabetic potential of *Gracilaria gracilis* SPs was investigated by assessing their effects on α-amylase, α-glucosidase, and pancreatic lipase activities as well as modulation of glucose absorption and uptake in rats’ small intestine and psoas muscle, respectively. The SPs were also characterized using chromatographic and spectroscopic analytical techniques in order to identify the bioactive compounds.

## 2 Materials and methods

### 2.1 Collection and preparation of macroalgal extract

The *Gracilaria gracilis* species were sourced from Wild Coast Abalone, East London, South Africa while identification of the species was carried out with the aid of Kelp (Pty) according to Olasehinde et al. [16].

Algal samples were rinsed thoroughly to washout the salt and sand particles. Pre-washed samples were then air-dried for 2 days before they were lyophilised in a freeze-dryer. Dried seaweed samples were then ground into a fine powder to increase their surface area [17]. About 100 g of seaweed material was defatted with n-hexane (500 ml) at room temperature with constant shaking for 24 h before the solvent was decanted, and the residue was air-dried overnight.

### 2.2 Extraction of sulphated polysaccharides

Crude SP-rich extracts were obtained through sequential cold and hot-water extractions followed by ethanol precipitation using the methods as described by Pengzhan et al. [18] and Olasehinde et al. [16]. In order to obtain the cold-water extract, 100 g of pre-treated dry algal material was extracted overnight with 500 ml of distilled water at room temperature. The resulting slurry was filtered through a Büchner funnel lined with a Whatman TG 100 Separating Gauze. The filtrate was transferred into a clean 2l Erlenmeyer flask, and 2 vol of absolute ethanol (EtOH) was added to the flask. After overnight precipitation at 4°C, the ethanol/water mixture was centrifuged for 5 min at 4000×*g*. The resulting supernatant was discarded, and the pellets recovered were lyophilised. The freeze-dried pellets were then ground into a powder (crude sulphated polysaccharides) using a mortar and pestle and then stored at 4°C until further use.

The hot-water extract was obtained by autoclaving 100 g of algae in 500 ml of distilled water for 15 min. The resulting slurry was cooled and then filtered through a Büchner funnel lined with a Whatman TG 100 Separating Gauze to separate the filtrate from the algal residue. The filtrate was then subjected to the same protocol used above in order to get the target extract.

### 2.3 Purification

The crude SP-rich extracts (soluble fraction) were purified via a GE AKTA Purifier 100 connected to a HiTrap DEAE Fast Flow column (Cytiva), according to Yu and Sun [19]. Briefly, the polysaccharide (100 mg) was dissolved in distilled water (5 ml) and centrifuged at 10 000 rpm for 10 min. The supernatant was filtered through a 0.2 μm syringe filter. The crude SP-rich extracts were then applied to a HiTrap DEAE FF column which was pre-equilibrated with distilled water. The column elution was carried out using NaCl solutions with increasing ionic strength from 0 to 2 M and at a flow rate of 1.25 ml/min. Then, eluents were pooled according to their total carbohydrate content and freeze-dried for other assay experiments.

### 2.4 Determination of total sugar content

The phenol–sulfuric acid method was used for the crude and purified SP-rich extracts to determine total carbohydrate content [20]. The 20, 40, 80, 120, 160, and 200 μg/ml standard glucose concentrations were prepared from a 1 mg/ml stock solution. Thereafter, 1 ml of 5% phenol solution and 5 ml of sulfuric acid were added to each corresponding concentration as well as 1 mg/ml of each SP-extract solution. After a 10 min incubation, the contents of each tube were vortexed and incubated for 20 min at 25–30°C. Then the absorbance was read at a wavelength of 490 nm and the concentration of sugar in the extracts was estimated from the standard curve of the varied glucose concentrations.

### 2.5 Determination of sulphate content

The sulphate content of the SP-extract samples was determined using the BaCl_2_-gelatin turbidity method [21]. In a typical procedure, 0.3% gelatine solution was prepared in hot water (60–70°C) stored overnight at 4°C. Two grams of BaCl_2_ were then dissolved in the gelatine solution and then allowed to stand for 2–3 h at 25°C. About 0.20 ml of polysaccharide solution (1 mg/ml) was added to 3.8 ml of 0.5 M HCl and 1 ml of BaCl_2_-gelatin reagent before equilibrating at room temperature for 10–20 min. The absorbance of suspension was measured at 360 nm using Cary 60 UV–Vis Spectrophotometer (Agilent) and the amount of released barium sulphate was obtained from a potassium sulphate (0–100 μg/ml) standard curve.

### 2.6 Functional group analysis

Fourier transform infrared (FT-IR) spectrophotometry was used to obtain the IR spectrum of the crude and purified SP-rich extracts’ functional groups. Approximately 1.5 mg of polysaccharide was ground with KBr and pressed to form pellet disc. The disc was then placed in the sample compartment before the spectra were obtained at 4000–400 cm^−1^ frequency range [22].

### 2.7 Analysis of monosaccharide composition

The analysis of the monosaccharide composition was carried out according to Honda et al. [23]. A mixture containing 100 μl of reconstituted sample, 50 μl of 0.3 M NaOH and 50 μl of PMP (1-pheny-3-methyl-5-pyrazolone) (0.5 M) was heated at 70°C for 60 min. After cooling to room temperature, the samples were neutralized with 50 μl of 0.3 M HCl and extracted 3 times with CHCl_3_ to remove underivatized PMP. Then, samples were analysed against a series of mixed sugar standards using a Water Acuity UPLC fitted with a photodiode array detector (Waters, Milford, USA). The system was coupled with a C18 (4.6 mm × 250 mm; 5 μm) column (Agilent Technologies, Santa Clara, CA, USA). The mobile phase was a solution containing (A) 20 mM phosphate buffer of varying pH levels (7.0. 7.5 and 8.0) and (B) acetonitrile (AcN) (15–18 %) in isocratic methods. The flow rate was set at 1 mL/min, the column temperature at 30°C, the injection volume at 10 μL and the detection at λ = 245 nm (DAD). The elution was performed in gradient as follows: 0 min–12% B, 35 min–17% B, 36 min–20% B, 45 min–20% B, 46 min–12% B, 65 min–12% B. The results were calculated using internal standard concentrations and response factors as mass percentages of each monomer, i.e., their anhydro forms, using polymerization factors of 0.9 for hexoses, 0.88 for pentoses, 0.89 for deoxy sugars and 0.907 for uronic acids.

### 2.8 Nuclear magnetic resonance (NMR) analysis

The samples were reconstituted in 600–700 μl of D_2_O and transferred to a new 5 mm capped NMR tube. A 400 MHz Bruker Avance Neo, fitted with a Broad Band Inverse 5 mm (BBI) probe head was utilised to collect the spectra. Each sample allocated 5 min temperature equilibration before the data was collected at 50°C. The 400 MHz 1H NMR spectra was collected first, followed by various H_2_O/HDO suppression experiments with 128 scans, which included a 5 s relaxation delay between excitation pulses and a 4 s acquisition time. The recorded data was processed on the Mnova 12 software package, which included phasing, baseline correction, referencing against the H_2_O/HDO signal, peak labelling, and appropriate line broadening.

### 2.9 *In vitro* antidiabetic activity screening

#### 2.9.1 α-Glucosidase inhibition

The ability of the SP-rich extracts and fractions to inhibit α-glucosidase activity *in vitro* was carried out according to the method described by Oboh and Ademosun [24] with slight modifications. Fifty microliters of each sample concentration (0.0625, 0.125, 0.25, 0.50, and 1 mg/ml) were incubated with an equal volume of α-glucosidase (1.0 U/ml) in phosphate buffer (100 mM, pH 6.8) at 37°C for 15 min. Thereafter, 100 μl of 5 mM *p*-nitrophenyl-α-d-glucopyranoside (pNPG) solution in phosphate buffer (100 mM, pH 6.8) was added to the reaction mixture before further incubation for 20 min at 37°C. The absorbance of the liberated *p*-nitrophenol was measured at 405 nm and the inhibitory activity was expressed as a percentage of the control using the formula below:

$$\%\;\text{inhibition}=\frac{\left(\text{Absorbance}\;\text{of}\;\text{control}-\text{Absorbance}\;\text{of}\;\text{sample}\right)}{\left(\text{Absorbance}\;\text{of}\;\text{control}\right)}\times 100$$

#### 2.9.2 α-Amylase inhibition

The ability of the seaweed extracts to inhibit α-amylase was investigated *in vitro* according to the method described by Shai et al. [25], with slight modifications. Fifty microliters of each sample concentration (0.0625, 0.125, 0.25, 0.50, and 1 mg/ml) or acarbose (ACR) was incubated with equal volumes of porcine pancreatic amylase (2 U/ml) in phosphate buffer (100 mM, pH 6.8) for 10 min at 37°C. After that, 50 μl of 1% starch solution in phosphate buffer (100 mM, pH 6.8) was added to the reaction mixture and incubated further at 37°C for 10 min. Then, 100 μl of the dinitrosalicylate (DNS) coloured reagent was added to the mixture and boiled for 10 min. Absorbance was read at 540 nm, and the inhibitory activity of the polysaccharide-rich extracts was expressed as a percentage of the control lacking the inhibitors.

#### 2.9.3 Pancreatic lipase inhibition

The ability of the SP-rich extract to inhibit pancreatic lipase activity *in vitro* was determined by the method of Kim et al. [26], with slight modifications. Fifty microliters of each sample or orlistat (standard drug) at concentration (0.0625, 0.125, 0.25, 0.50, and 1 mg/ml) were equilibrated with 84.5 μl of Tris buffer (100 mM Tris–HCl and 5 mM CaCl_2_, pH 7.0), 20 μl of porcine pancreatic lipase (2.5 mg/ml in 10 mM MOPS (morpholine propane sulphonic acid) and 1 mM EDTA (pH 6.8) at 37°C for 15 min. Then 5 μl of 10 mM *p*-NPB (*p*-nitrophenyl butyrate in dimethyl formamide) was added to the reaction mixture and further incubated for 30 min at 37°C. Absorbance was then read at 405 nm, and the inhibitory activity of the samples was expressed as percentage of the control lacking the inhibitors.

#### 2.9.4 Glucose uptake by yeast cells

The effect of the crude and purified SP-rich extracts on glucose uptake by yeast cells was carried out according to the protocol of Pitchaipillai and Ponniah [27]. Different concentrations of the SP-rich extracts and purified fractions were dissolved in 1 ml of distilled water containing 25 mM glucose. The resulting solution was incubated for 10 min at 37°C. Thereafter, 100 μl of 1 % yeast suspension was added, vortexed and incubated for 60 min at 37°C. Then, glucose concentration of the solution was determined with dinitro salicylic acid method (DNS), and the % glucose uptake was calculated using the formula:

$$\%\;\text{Glucose}\;\text{uptake}=\frac{\left(\text{Absorbance}\;\text{of}\;\text{control}-\text{Absorbance}\;\text{of}\;\text{sample}\right)}{\left(\text{Absorbance}\;\text{of}\;\text{control}\right)}\;X\;100$$

### 2.10 *Ex vivo* antidiabetic activity screening

#### 2.10.1 Animals

Twelve (12) male Sprague-Dawley rats with 180 ± 10.12 g average body weight were collected from the Biomedical Resource Unit (BRU), University of KwaZulu-Natal, Durban, South Africa. The animals were denied food but not water for 12 h and then put to sleep with isofor anaesthesia. The animals’ gastrointestinal tract (GIT) and psoas muscle portions were harvested after dissection for intestinal glucose absorption and muscle glucose uptake studies, respectively. The animals were handled in cognisance to the rules and regulations of the Animal Research Ethics Committee of the University of KwaZulu-Natal, Durban, South Africa (Ethical Approval Number: AREC/00002347/2021).

#### 2.10.2 Measurement of glucose absorption in rat intestinal jejunum

This assay was carried out by measuring the concentrations of glucose in an incubation mixture consisting of the pre-cut rat jejunum and test samples according to Hassan et al. [28] and as modified by Chukwuma et al. [29]. Briefly, jejunal segments of the harvested small intestine were cut into smaller pieces of 5 cm and then inverted to expose the inner lumen, cleaned with 2 ml of Kreb's buffer (118 mM NaCl, 5 mM KCl, 1.328 mM CaCl_2_·2H_2_O, 1.2 mM KH_2_PO_4_, 1.2 mM MgSO_4_ and 25 mM NaHCO_3_) in an antiseptic syringe. The pieces then placed in an incubation tube containing 8 ml of Krebs buffer, 11.1 mM glucose and varying concentrations of SP-containing extracts. Then 1 ml mixture was taken from each incubation tube before and after the 2 h incubation time in a Steri-Cult CO_2_ incubator (Nuaire, Doncaster, UK) with 5 % CO_2_, 95% oxygen and 37°C settings. Subsequently, the amount of glucose in the samples was determined using an Automated Chemistry Analyzer (Labmax Plenno, Labtest Inc., Lagoa Santa, Brazil). The extent of intestinal glucose absorption in the presence and absence of the test samples was calculated using the following formula:

$$Intestinal\;glucose\;absorption\;\left(\frac{mg}{cm}\;jejunum\right)=\frac{\left(\;\text{GC}1-\text{GC}2\;\right)}{\left(\text{lenght}\;\text{of}\;\text{jejunum}\;\left(\text{cm}\right)\right)}$$

where GC1 & GC2 are the glucose concentrations (mg/dl) before and after the incubation, respectively.

#### 2.10.3 Measurement of glucose uptake by rat psoas muscles

This assay was done using the procedure described by Abdel-Sattar et al. [30] and as modified by Chukwuma et al. [29]. The harvested psoas muscle was cut into small chunks of the same weight (500 mg). Each chunk was incubated in 8 ml of Krebs buffer, pre-mixed with 11.1 mM glucose (control) and varying concentration of the samples or 0.5 mg/ml or metformin (positive control). The mixture was transferred to an incubator (as mentioned earlier) with 5% CO_2_, 95% oxygen at 37°C. Before and after the incubation, 1 ml solution was collected from each incubation tube and the glucose concentration was measured using the Chemistry Analyzer equipment as mentioned earlier. The level of glucose uptake by the rats’ psoas muscle in the presence or absence of the test samples was calculated using the following formula:

$$\text{Muscle}\;\text{glucose}\;\left(\frac{\text{mg}}{g}\text{tissue}\right)=\frac{\left(\text{GC}1-\text{GC}2\right)}{\left(\text{Weight}\;\text{of}\;\text{muscle}\;\text{tissue}\;\left(g\right)\right)}$$

where GC1 and GC2 are glucose concentrations (mg/dl) before and after incubation, respectively.

## 3 Results

### 3.1 Chemical composition of sulphated polysaccharides (SPs)

The percentage yields of the hot and cold aqueous SP-rich extracts derived from *Gracilaria gracilis* is displayed in Table 1. The hot aqueous SP-rich extract exhibits a significantly higher percentage yield as compared to the cold aqueous SP-rich extract. While the *Gracilaria gracilis* hot aqueous extract had statistically higher total sugar content than the cold algae extract, the total sulphate content of the latter SP-rich extract was significantly higher that of the former extract.

**Table 1:** Chemical composition of sulphated polysaccharide-rich extracts derived from *Gracilaria gracilis*.

Extraction method	Percentage yield (%)	Total sugar content (mg/g)	Total sulphate content (mg/g)
Hot aqueous SP	8.23 ± 0.24^a^	0.26 ± 0.01^a^	0.05 ± 0.01^a^
Cold aqueous SP	7.82 ± 0.85^b^	0.095 ± 0.01^b^	0.19 ± 0.01^b^

Data are presented as mean ± SD. ^ab^Values within a column with different superscript letters are significantly different from each other (*p* < 0.05). SP, sulphated polysaccharides.

### 3.2 FT-IR analysis

**Fig. 1:**
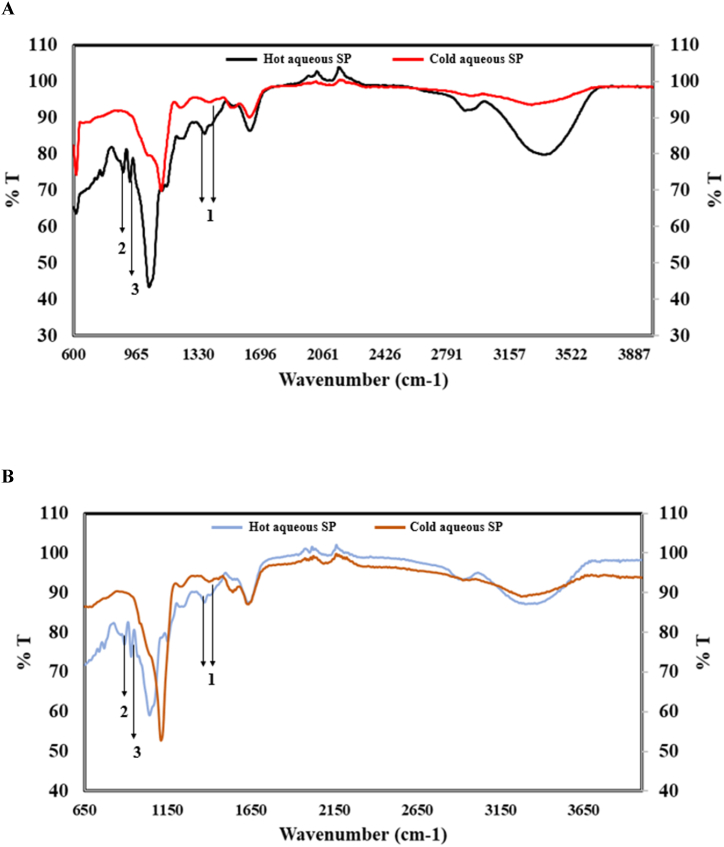
FT-IR analysis of crude (**A**) and purified (**B**) hot and cold aqueous SP-rich extracts from *G. gracilis. Peak labels:***1-** The presence of a sulphate functional group (S=O), **2**- Characteristic presence of d-galactose, **3**- Vibrations associated with 3,6-anhydrogalactose.

Fig. 1A and 1B revealed the result of the functional groups detected in the crude and purified SP-rich seaweed extracts. The vibration displayed for the crude and purified SP-rich extracts at 3500-3200 cm^−1^ is linked to the presence of O–H and C–H functional groups. Peaks at 891 and 931 cm^−1^ were unique to the crude hot aqueous extract while the signal obtained at 891 cm^−1^ shows that the crude hot aqueous extract contains a C–O–S stretch indicating the presence of d-galactose. The peak at 931 cm^−1^ was assigned to C–O–C vibration of 3,6-anhydrogalactose. Consequently, these results indicate that the crude hot aqueous extract is a sulphated polysaccharide with mainly α-glycosidic bonds. Moreover, both crude SP-rich extracts showed strong intensities at 1350–1450 cm^−1^, suggesting the presence of S = O groups.

### 3.3 Monosaccharide composition

The results in Table 2 showed *Gracilaria gracilis* crude and purified SP-rich extracts’ monosaccharide composition. The LC-MS analysis revealed the presence of glucose, mannuronic acid, galactose, arabinose, xylose, and glucuronic acid. The glucose concentration in the crude extracts were higher than those in the purified aqueous extracts. While mannuronic acid was identified only in the crude cold extract, the carbohydrate concentration (81.3 mg/l) was in the range of glucuronic acid measured in both the crude and the purified seaweed extracts (81.6–82.8 mg/l). Although xylose was present in the crude and purified extracts the recorded concentrations (0.5–2.8 mg/l) of the sugar were lower than that of galactose and arabinose (6.9–26.0 mg/l).

**Table 2:** Monosaccharide composition of *Gracilaria gracilis* seaweed extracts.

Extracts	Monosaccharide units (mg/l)						
	Glu	ManA	GluA	Gal	Ara	Xyl	Total
Crude							
Hot aqueous	102.7	–	82.8	17.1	15.1	2.8	202.62
Cold aqueous	106.6	81.3	81.7	11.9	6.9	0.5	288.9
Purified							
Hot aqueous	59.9	–	81.6	22.4	13.0	1.4	178.3
Cold aqueous	73.5	–	82.8	26.0	17.8	2.7	202.8

*Glu, Glucose; **ManA,** Mannuronic acid; **GluA**, Glucuronic acid; **Gal**, Galactose; **Ara**, Arabinose; **Xyl**, Xylose.

### 3.4 NMR analysis of purified sulphated polysaccharides

**Fig. 2:**
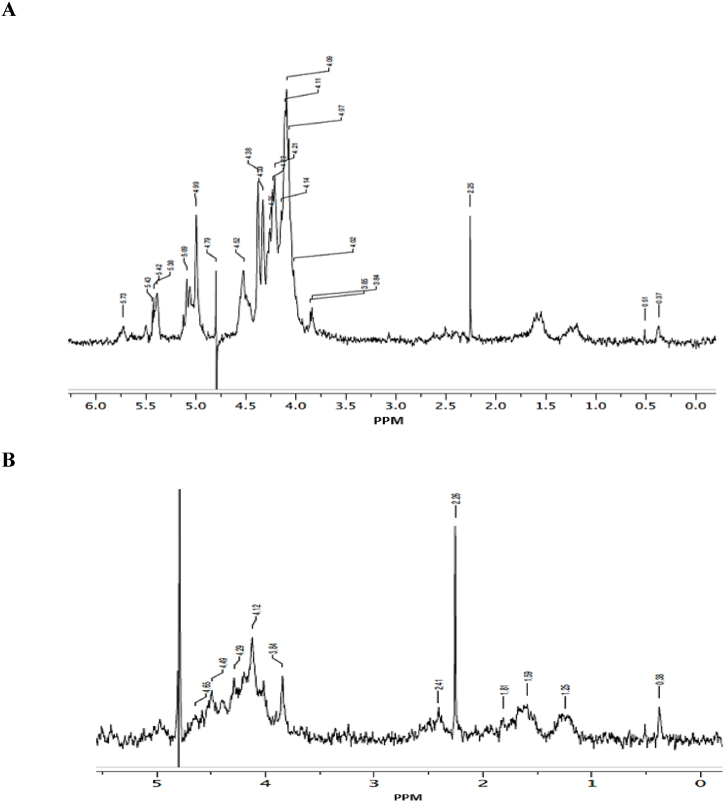
H^1^NMR analysis of hot (A) and cold (B) aqueous SP-rich extracts from *G. gracilis*.

The results in Fig. 2A shows the H^1^NMR spectrum of the purified hot aqueous SP derived from *G. gracilis*. The signal from the α anomeric proton at δ 5.09 was assigned to 3,6-α-l-anhydrogalactose while the signal at δ 4.52 was attributed to β-d-galactose (G). The signal observed at δ 4.38 was assigned to H-1 of β-d-galactose linked to α-l-galactose-6-sulphate, and the signal at δ 5.38 was attributed to anomeric proton of 6-O-sulphate-l-galactopyranose. The spectrum also revealed resolution of signals at ∼δ 3.84 which shows the methylation pattern with protons of methyl groups attached to the O-6 of β-d-galactose. The resulting spectra in Fig. 2B shows the H^1^NMR spectrum of the purified cold aqueous SP extracts derived from *G. gracilis*. The signal at δ 4.49–4.65 was attributed to β-d-galactose (G). The signal observed at ∼ δ 4.29 was assigned to H-1 of β-d-galactose linked to α-l-galactose-d-sulphate while the resonance at ∼δ 1.59, may be attributed to methyl protons of the cyclic pyruvate acetal as 4,6-O-(1-carboxyethylidene) group. This spectrum also revealed a methylation pattern with resolution of signals at ∼

δ 3.84 for protons of methyl groups attached to O-6 of β-d-galactose.

### 3.5 Antidiabetic potential of sulphated galactans

#### 3.5.1 α-Glucosidase inhibition assay

**Fig. 3:**
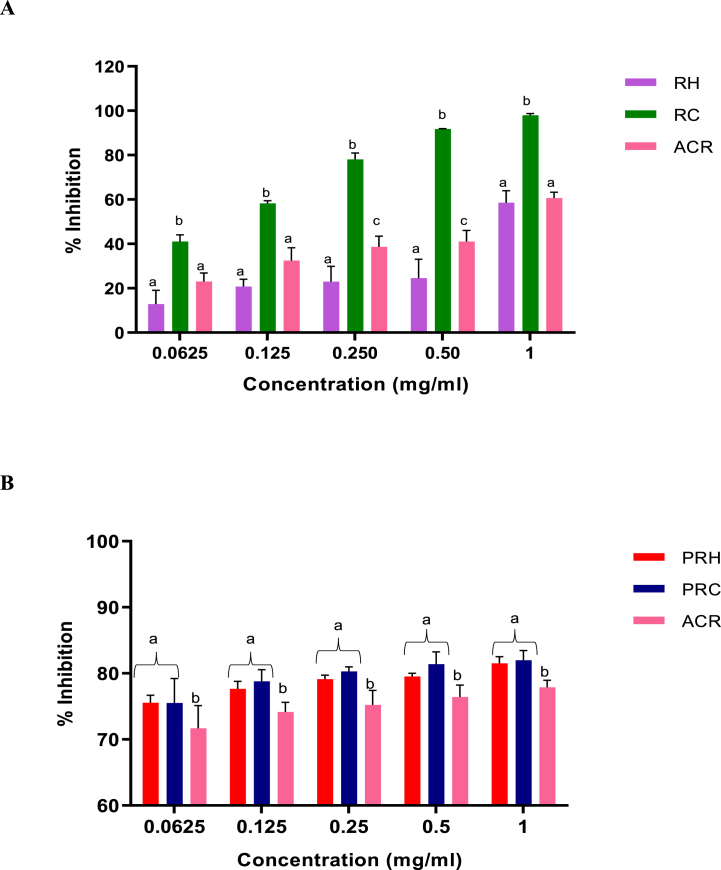
α-glucosidase inhibitory activities of crude (**A**) and purified (**B**) hot and cold aqueous SP-rich extracts derived from *G. gracilis* seaweed. Data = mean ± SD; n = 3. ^abc^ Bars with different letters for a given concentration are significantly (p < 0.05) different from each other. RH: crude hot SP-rich extracts, RC: cold SP-rich extracts, PRH: purified hot SP-rich extracts, PRC: purified cold SP-rich extracts, ACR: acarbose.

In Fig. 3A, the enzyme inhibition by all the SP-rich crude extracts including the standard drug acarbose (ACR) increased in a dose-dependent manner. The cold aqueous SP-rich extract (RC) had the highest percentage of inhibition (97.98 %) at 1 mg/ml concentration compared to the hot aqueous SP-rich extract (RH) (58.59 %) and ACR (60.66 %). While RH (IC_50_ = 0.83 mg/ml) and RC (IC_50_ = 0.15 mg/ml) due to their lower IC_50_ values as seen in Table 3 displayed significantly (*p* < 0.05) more potent inhibitory activity against α-glucosidase than ACR (4.42 mg/ml), the activity of the latter extract was better than the former. In Fig. 3B, there was no significant difference (*p* > 0.05) in the enzyme inhibition between the purified SP-rich extracts ate the test concentrations. However, the relative 0.07 mg/ml and 0.08 mg/ml IC_50_ values for PRH and PRC extracts in Table 4 suggest their greater potencies than ACR (IC_50_ = 4.42 mg/ml) standard compound.

**Table 3:** IC_50_ values of *G. gracilis* derived crude SP-rich extracts *in vitro* bioactivities (mg/ml).

Activities	Cold aqueous	Hot aqueous	Acarbose	Orlistat
α-Glucosidase	0.15 ± 0.01^a^	0.83 ± 0.28^b^	4.42 ± 0.45^c^	–
α-Amylase	2.02 ± 0.01^a^	0.59 ± 0.01^a^	0.14 ± 0.01^b^	–
Pancreatic lipase	0.17 ± 0.01^a^	0.03 ± 0.05^b^	–	0.19 ± 0.01^a^
Glucose uptake	1.17 ± 0.19^a^	0.01 ± 0.01^b^	0.43 ± 0.03^c^	–

Data are presented as mean ± SD. ^ab^Values within a row with different superscript letters are significantly different from each other (*p* < 0.05).

**Table 4:** IC_50_ values of purified sulphated polysaccharide-rich extracts bioactivities (mg/ml).

Activities	Cold aqueous	Hot aqueous	Acarbose	Orlistat
α-Glucosidase	0.08 ± 0.02^a^	0.07 ± 0.02^a^	4.42 ± 0.45^b^	–
α-Amylase	0.17 ± 0.06^a^	0.31 ± 0.19^b^	0.14 ± 0.01^a^	–
Pancreatic lipase	0.24 ± 0.01^a^	0.51 ± 0.08^b^	–	0.52 ± 0.07^b^
Glucose uptake	0.24 ± 0.09^a^	0.18 ± 0.01^b^	0.43 ± 0.03^c^	–

Data are presented as mean ± SD. ^ab^Values within a row with different superscript letters are significantly different from each other (*p* < 0.05).

#### 3.5.2 α-Amylase inhibition assay

**Fig. 4:**
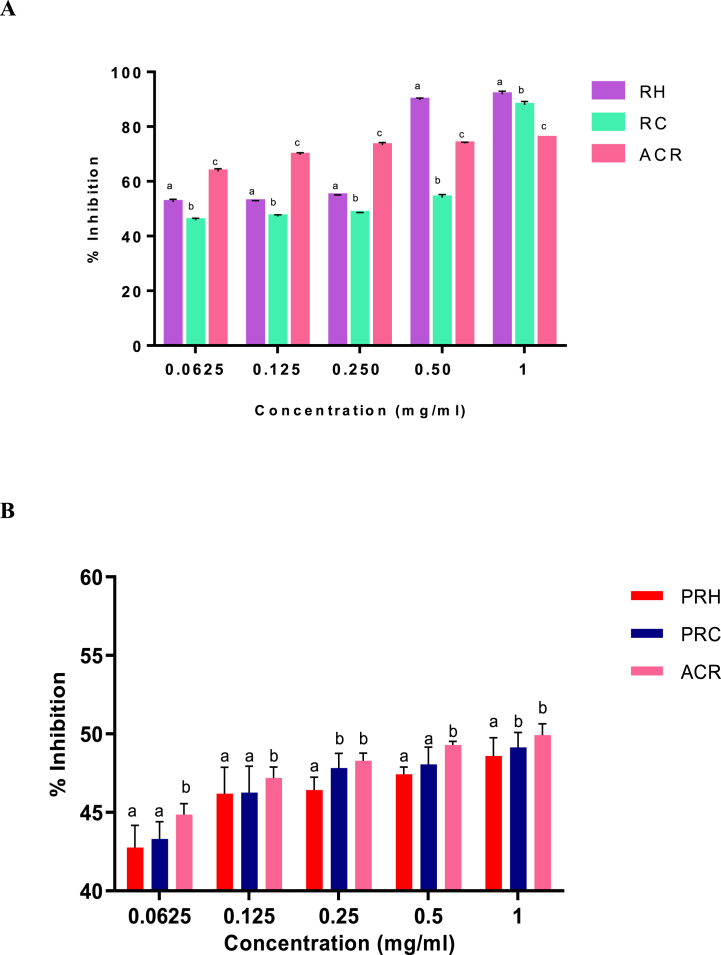
α-amylase inhibitory activities of crude (**A**) and purified (**B**) hot and cold aqueous SP-rich extracts derived from red seaweed. Data = mean ± SD; n = 3. ^abc^Bars with different letters for a given concentration are significantly (p < 0.05) different from each other. RH: crude hot SP-rich extracts, RC: cold SP-rich extracts, PRH: purified hot SP-rich extracts, PRC: purified cold SP-rich extracts, ACR: acarbose. (For interpretation of the references to colour in this figure legend, the reader is referred to the Web version of this article.)

The inhibitory effects of *G. gracilis* crude extracts on α-amylase enzyme activity is shown in Fig. 4A. Although the two crude extracts (RH and RC) had significantly lower α-amylase inhibition than acarbose at 0.0625–0.5 mg/ml, the extracts’ activities were better than those of the drug at 1.0 mg/ml the data for crude extracts, as presented in Table 3, indicate that the RH with IC_50_ = 0.59 mg/ml has better α-amylase inhibitory activity than the RC with 2.02 mg/ml IC_50_. In Fig. 4B, there was no significant difference in the inhibitory potential between the purified SP-rich extracts at 0.0625, 0.125 and 0.5 mg/ml concentrations. However, the IC_50_ values as seen in Table 4 showed that PRC (IC_50_ = 0.17 mg/ml) exhibited α-amylase inhibitory activities statistically similar with ACR (IC_50_ = 0.14 mg/ml) but significantly (*p* < 0.05) higher than those of PRH (IC_50_ = 0.31 mg/ml).

#### 3.5.3 Pancreatic lipase inhibition assay

**Fig. 5:**
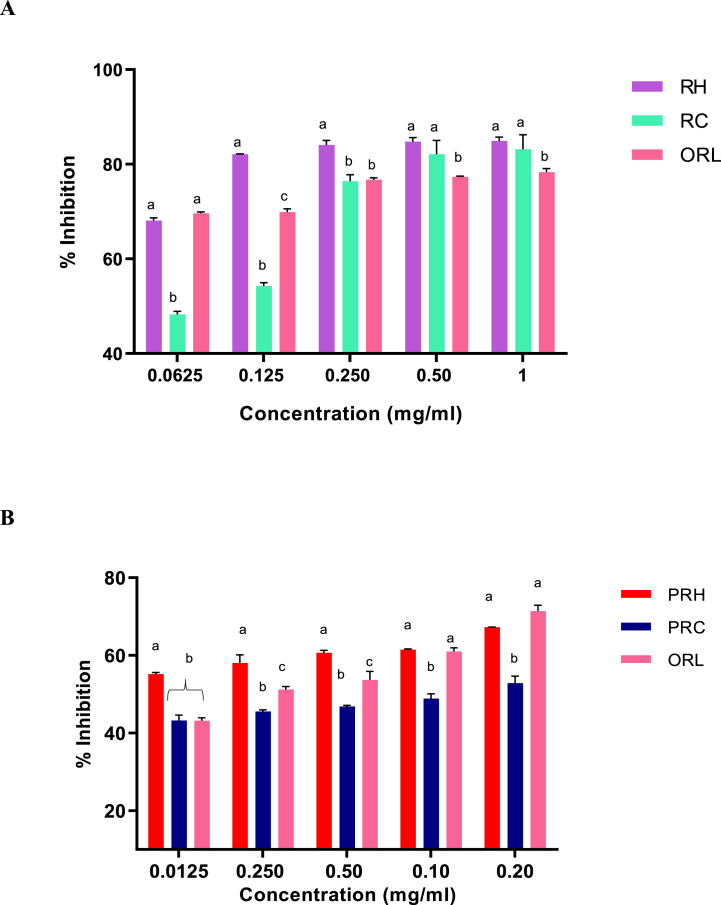
Pancreatic lipase inhibitory activities of crude (**A**) and purified (**B**) hot and cold aqueous SP-rich extracts derived from *G. gracilis seaweed*. Data = mean ± SD; n = 3. ^abc^Bars with different letters for a given concentration are significantly (p < 0.05) different from each other. RH: crude hot SP-rich extracts, RC: cold SP-rich extracts, PRH: purified hot SP-rich extracts, PRC: purified cold SP-rich extracts, ORL: orlistat.

Fig. 5Aand 5B shows the inhibition effects of RH and RC extracts on pancreatic lipase, respectively. At 0.0625 and 0.125 mg/ml concentrations, both the crude SP-rich extracts significantly (*p* < 0.05) outperformed the inhibitory activity of orlistat. The IC_50_ values as seen in Table 3 showed that RH (IC_50_ = 0.03 mg/ml) exhibited significantly (*p* < 0.05) higher inhibitory effect on lipase, compared to RC (IC_50_ = 0.17 mg/ml) and orlistat (IC_50_ = 0.19 mg/ml). There was no significant difference (*p* > 0.05) in the inhibition between the PRH (IC_50_ = 0.51 mg/ml) and orlistat (IC_50_ = 0.52 mg/ml) in Table 4. However, the PRC (IC_50_ = 0.24 mg/ml) had a significantly (*p* < 0.05) higher inhibitory activity than the other two treatments.

#### 3.5.4 Glucose uptake by yeast cells

**Fig. 6:**
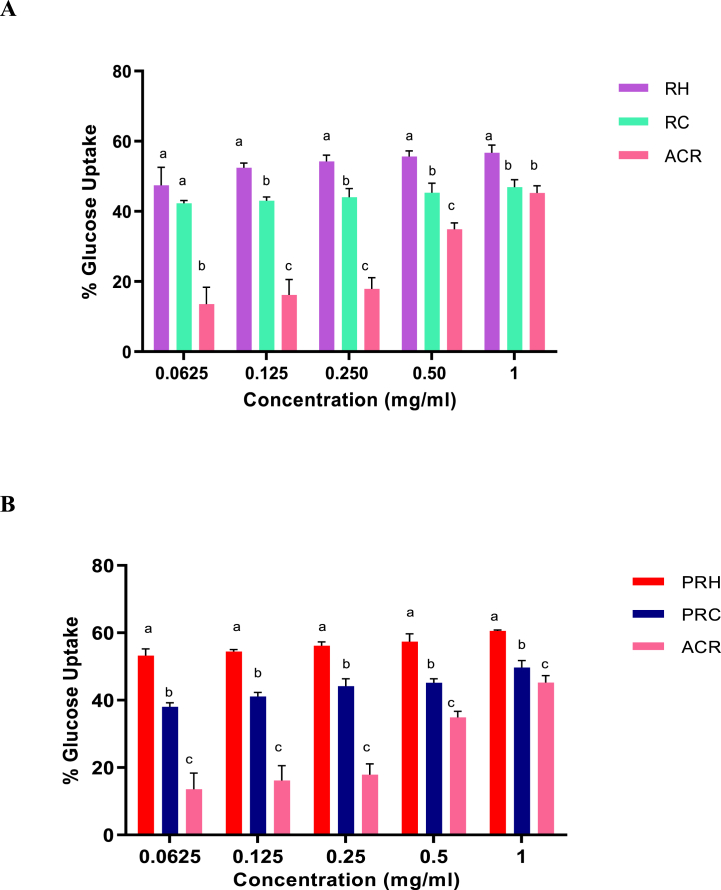
Glucose uptake by yeast cells by crude (**A**) and purified (**B**) hot and cold aqueous SP-rich extracts derived from *G. gracilis seaweed*. Data = mean ± SD; n = 3. ^abc^Bars with different letters for a given concentration are significantly (p < 0.05) different from each other. RH: crude hot SP-rich extracts, RC: cold SP-rich extracts, PRH: purified hot SP-rich extracts, PRC: purified cold SP-rich extracts, ACR: Acarbose.

In Fig. 6A, treatment with the *G. gracilis* crude SP-rich caused significant increase in glucose uptake by the yeast cells. Although the RH extract produced higher sugar uptake than the RC extract, both extracts displayed properties that were higher than those of acarbose at the test concentrations (0.0625–1.0 mg/ml). Interestingly, a similar trend was observed for the purified extracts as seen in Fig. 6B. There was a significant difference (*p* < 0.05) in the stimulation of glucose uptake between the purified SP extracts at all concentrations where PRH had a better stimulatory activity than PRC. Based on the results presented in Table 4, PRH (IC_50_ = 0.18 mg/ml) had a higher glucose uptake activity than PRC (IC_50_ = 0.24 mg/ml) and ACR (IC_50_ = 0.43 mg/ml).

### 3.6 *Ex vivo* antidiabetic potential of *G. gracilis*

#### 3.6.1 Effects of SP-rich extracts on glucose absorption in rat jejunum

**Fig. 7:**
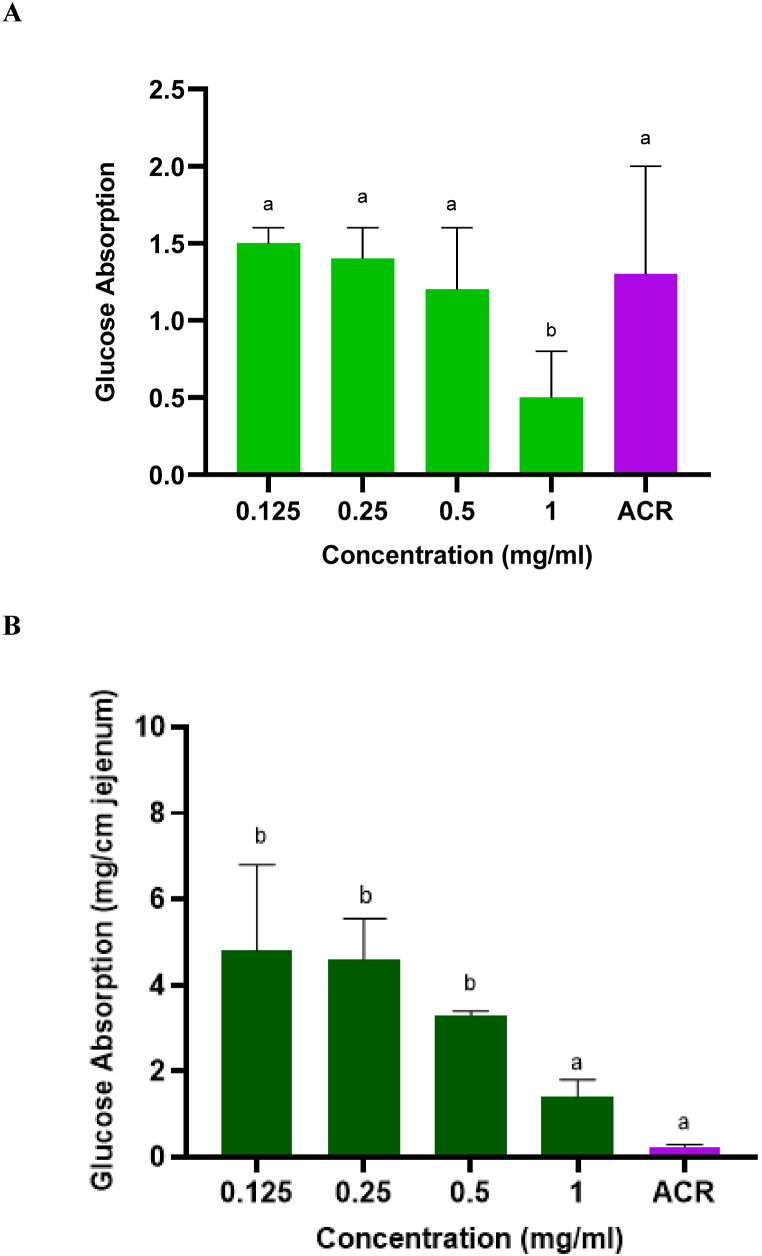
The effects of *G. gracilis* crude (**A)** and purified (**B**) SP-rich extracts on glucose absorption in isolated rat jejunum. Data are presented as mean ± SD. Data = mean ± SD; n = 3. ^ab^Bars with different letters are significantly (p < 0.05) different from each other. ACR: acarbose (1 mg/ml).

The extent of glucose absorption by rat isolated jejunum in the presence of crude SP extracts is shown in Fig. 7A. At 1 mg/ml the amount of glucose absorbed by the crude SP extract was at the lowest and was significantly (*p* < 0.05) lower than those observed with ACR. In Fig. 7B incubation with the purified SP extract significantly (*p* < 0.05) inhibited the absorption of glucose by the intestinal tissue with the seaweed extracts showing dose-dependent activity. The amount of glucose absorbed was the lowest for ACR. However, there was no significant difference (*p* > 0.05) between the purified SP extract and ACR at highest experimental concentration (1 mg/ml).

#### 3.6.2 Effects of SP on glucose uptake in rat psoas muscle

**Fig. 8:**
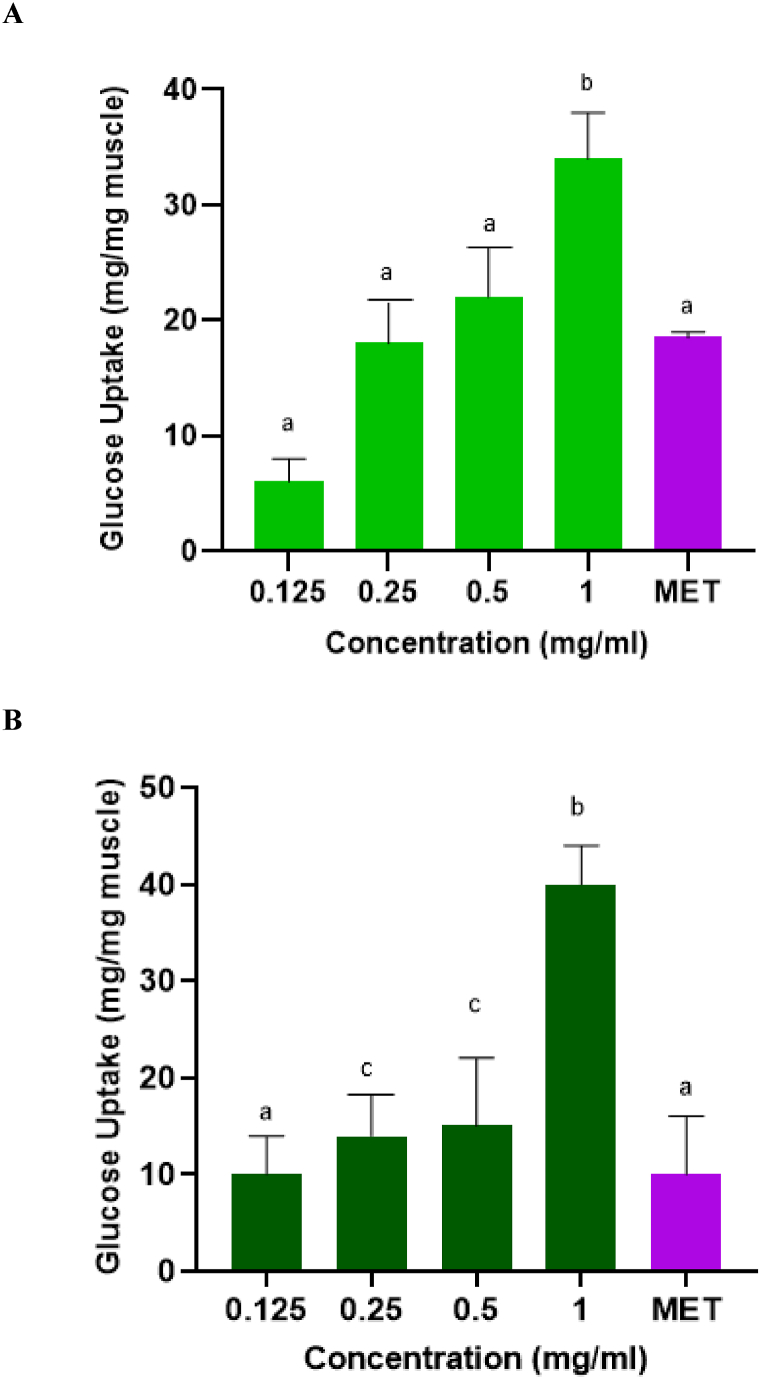
The effects of *G. gracilis* crude (**A)** and purified (**B**) SP-rich extracts of glucose uptake in isolated rat psoas muscle. Data are presented as mean ± SD. Data = mean ± SD; n = 3. ^ab^Bars with different letters are significantly (p < 0.05) different from each other. MET: metformin (1 mg/mL).

Fig. 8A depict a significant (*p* < 0.05) increase in glucose uptake in muscle tissue incubated with the crude SP extracts. The stimulation of glucose uptake increased with increasing concentration. Despite this, there was no significant difference (*p* > 0.05) in the glucose uptake between metformin and the crude SP extract at lower concentrations (0.125–0.5 mg/ml). However, at 1 mg/ml, the SP extract showed significantly (*p* < 0.05) better glucose uptake than the standard drug. In Fig. 8B, the effect metformin treatment on muscle glucose uptake was indistinguishable from those observed in the seaweed purified extract at 0.125 mg/ml. However, the glucose uptake effect of the purified extract, especially at 1 mg/ml, was significantly (*p* < 0.05) higher than those of the metformin clinical drug.

## 4 Discussion

Seaweeds and more importantly, seaweed polysaccharides have displayed potential biological applications as functional foods, cosmeceuticals, nutraceuticals, and pharmaceutical products [31]. Algal polysaccharides can be broadly classified into two groups namely: sulphated and non-sulphated. In natural environments, sulphated polysaccharides tend to be a diverse group of bioactive compounds [32]. Their diversity stems from the variations in their carbohydrate backbone, the position of their sulphate groups and their degree of sulphation [33]. In order to study these crucial phytoconstituents in plant species, appropriate extraction methods must be employed to separate them from other components that may be of no therapeutic importance. The current study observed a significant difference between extraction methods in terms of yield and total sulphate content. The hot aqueous displayed a higher yield than the cold aqueous extract as seen in Table 1. A similar trend was observed in a previous study by Souza et al. [34], where the exhaustive aqueous extraction of SPs from *G. birdiae* at high temperatures (90°C) produced samples with higher yield than those obtained via cold extraction. Maciel et al. [35] also revealed that the lower yield from *G. birdiae* during cold extraction might be attributed to low temperatures. Extraction temperature plays a vital role on the yield of seaweed materials. An effect that has been further supported by various other findings [36]. Moreover, in this study, the sulphate content in *G. gracilis* hot water extract SPs was lower than those of the cold extract. A study by Mazumder et al. [37] attributed the high sulphate content (0.23 mg/ml (11.7%)) of *Gracilaria corticata* SPs extract to cold extraction. According to this study, low temperatures may result in lower SP yield, but they could allow the extraction of complex polysaccharides with higher sulphate concentration.

Using infrared light to scan the samples, Fourier Transform Infrared Spectroscopy (FT-IR) is an analytical technique for identifying organic, inorganic, and polymeric materials [38]. FT-IR analysis is important for identifying and describing unidentified components, locating additions, detecting impurities in a substance, and determining decomposition and oxidation [39]. The spectrum from the FT-IR analysis of both the crude and purified SP-rich extracts showed a typical spectrum vibration associated with sulphated polysaccharides and more specifically, sulphated galactans [40]. The presence of 3,6-anhydrogalactase was noted. Although 3,6-anhydrogalactose has yet to be studied as a potential antidiabetic agent, findings describing its use as an anti-inflammatory agent have produced promising results. Consequently, there are indications that the bioactivity of algal polysaccharides can be attributed to the presence of sulphate functional groups on the sugar backbone [41].

These monosaccharide profile contained a significant amount of sugar present in seaweed. Galactose was present, which is the primary sugar in the sulphated polysaccharides that several species in the *Gracilariales* order produce [42]. It has yet to be fully determined if the sugars present in *G. gracilis* is linked to the antidiabetic potential of the species. However, it is generally established that the sulphate content, location of sulphate groups within the SP backbone and sugar composition in the polysaccharide all directly affect the antioxidant activity of SPs [43,44].

NMR spectroscopy was carried out to predict the structure of any regular and complex polysaccharides [45]. The H^1^NMR spectra of polysaccharides comprise of well-resolved signals from 4.4 to 5.5 ppm (Fig. 2A and B). The outcomes presented in the current study are supported by those found in previous literature [15,46].

Regardless of the recent advancements in the search for effective therapeutic agents for the management of T2DM, the number of individuals being diagnosed each year is steadily increasing [47]. As the primary producers of food and energy in most aquatic habitat, seaweed tend to serve as the biggest producers of antidiabetic compounds and has attracted interest from the pharmaceutical sector in the management of diabetes [13]. The primary source of the glucose molecules needed by the human body to produce energy is dietary carbs [48]. Catabolic digestive enzymes, which hydrolyse complex carbohydrate polymers into smaller monosaccharide units, aid in this process [49]. Amylase and glucosidase are two of these enzymes that work in the digestive tract to increase the amount of glucose delivered into the bloodstream through intestinal absorption [50]. As a result, the inhibition of these enzymes has been investigated as a possible treatment of diabetes and associated complications.

In this study, the SP-rich extracts superior α-glucosidase inhibition may be attributed to synergistic effects of the SPs. In support of the present findings, Liao et al. [51] reported that SP-rich extracts from *Gracilaria lemaneiformis* algal specie similarly showed considerable α-glucosidase inhibition when compared with acarbose standard drug. Even though the inhibition of α-amylase decreases maltose and glucose production from starch, excessive inhibition of the enzyme could lead to the accumulation of undigested starch, which fermentation by the intestinal flora may, in turn, causes gastrointestinal issues [52]. Hence, partial inhibition of α-amylase may be preferred as seen in Fig. 4 and Table 4. As observed in this study, the inhibitory properties of both crude and purified SP-rich extracts may indicate that SP-rich extracts derived from *G. gracilis* may have the potential ability to aid the management of T2DM through partial α-amylase inhibition.

Almost 70% of patients with T2DM are obese, hence the relationship between obesity and T2DM can be considered interdependent, since being obese significantly increases the chances of T2DM [53]. Reducing excessive energy intake by lowering intestinal absorption of dietary fat is a frequent strategy for treating obesity [54]. Due to their fewer adverse effects when compared to synthetic medications, natural bioactivities such as sulphated polysaccharides that suppress pancreatic lipase activity have been a prominent therapeutic target in this area [55]. Balasubramaniam et al. [56] carried out a study on the inhibitory activities of three tropical red seaweed viz., *K. alvarezii*, *K. striatus* and *Euchema denticulatum*. All three seaweeds displayed significant lipase inhibitory activities. Similar findings were demonstrated by O'Connor et al. [57], who tested the inhibitory effects of soluble fibres on calf pre-gastric lipase. Likewise, in the present study, *G. gracilis* crude and purified SP-rich extracts showed pancreatic lipase inhibition for the first time (Fig. 5). Consequently, this effect of the SP-rich extracts on pancreatic lipase activity suggests that *G. gracilis* may exhibit strong anti-obesogenic potentials and could be explored as an alternative therapy to limit diabetes-linked hyperlipidaemia.

Before determining the ability of a bioactive compound as a potential stimulant of glucose uptake in muscle tissue *ex vivo*, researchers prefer testing its’ abilities *in vitro.* Pitchaipillai and Ponniah [27], associated yeast glucose uptake properties with antidiabetic potentials. Thus, the ability of the SP-rich extracts to enhance glucose uptake in yeast cells (Fig. 6A and B) may suggest their capacity to elicit similar biological activity in peripheral tissues under hyperglycaemic conditions.

The treatment of T2DM involves a multi-faceted approach, in which both the breakdown of complex polysaccharides into simple sugars, glucose absorption and the uptake of said sugars need to be managed [58]. Studies have shown that chemical agents with the ability to inhibit glucose absorption in the intestine may present an explorable diabetes treatment regime that could help lower blood glucose levels [59]. The results displayed in Fig. 7 coincide with the inhibitory effects on carbohydrate-hydrolysing activities as seen in this study. Since the inhibition of α-glucosidase and α-amylase results in a decreased production of glucose, the absorption of the sugar will also be limited. As seen in Fig. 8, higher concentrations of the crude and purified SP extracts were comparably better than metformin at stimulating glucose uptake. Metformin reduces blood sugar levels by inhibiting gluconeogenesis in the liver and promoting glucose uptake and utilization in peripheral tissues. As observed in this study, the superior effect of the extracts could reflect better effectiveness in carrying the same bioactivity under similar physiological conditions as reported previously by Polianskyte-Prause et al. [60].

## 5 Conclusion

With the occurrence of T2DM on the increase, efforts are being made to find natural therapies that can manage hyperglycaemia and its side-effects. The SP extracts from *G. gracilis* displayed various characteristics and peaks at wavelength that indicated the presence of sulphated galactans. The monosaccharide composition showed that SP-rich extracts from *G. gracilis* contained galactose, a functional constituent of the polysaccharide backbone. Further analysis using NMR also confirmed for the presence of galactose sub-units. The result of this study demonstrates that *Gracilaria gracilis* SP-rich extracts inhibit enzymes involved in carbohydrate and lipid digestion as well as stimulate glucose uptake in yeast cells, psoas muscle while limiting glucose absorption in the intestinal tissue. Although it appears that sulphate functional group in the seaweed SP may be responsible for the inhibitory activities of the seaweed extracts, there is the need to further validate the anti-hyperglycaemic efficacy of this SPs via *in vivo* studies.

## Data availability statement

The data in support this research findings will be made available on a reasonable request.

## Ethical statement

This study protocol was approved by the Animal Research Ethics Committee of the University of KwaZulu-Natal, Durban, South Africa (Ethical Approval Number: AREC/00002347/2021).

## CRediT authorship contribution statement

**Leah R. Pillay:** Writing – review & editing, Writing – original draft, Methodology, Investigation, Funding acquisition, Formal analysis, Data curation. **Tosin A. Olasehinde:** Writing – review & editing, Writing – original draft, Validation, Supervision, Investigation, Funding acquisition, Formal analysis, Conceptualization. **Kolawole A. Olofinsan:** Writing – review & editing, Visualization, Validation, Software, Methodology, Investigation, Formal analysis. **Ochuko L. Erukainure:** Writing – review & editing, Validation, Methodology, Investigation, Data curation. **Md. Shahidul Islam:** Writing – review & editing, Validation, Supervision, Methodology, Formal analysis, Data curation, Conceptualization. **Ademola O. Olaniran:** Writing – review & editing, Validation, Supervision, Resources, Project administration, Methodology, Funding acquisition, Formal analysis, Data curation, Conceptualization.

## Declaration of competing interest

The authors declare that they have no known competing financial interests or personal relationships that could have appeared to influence the work reported in this paper.
