# Supplementary figures and images for: Antidiabetic potentials of crude and purified sulphated polysaccharides isolated from Gracilaria gracilis, a seaweed from South Africa
Source: Heliyon. 2024 Aug 2;10(15):e35729. doi: 10.1016/j.heliyon.2024.e35729 (PMC11336833; doi:10.1016/j.heliyon.2024.e35729)

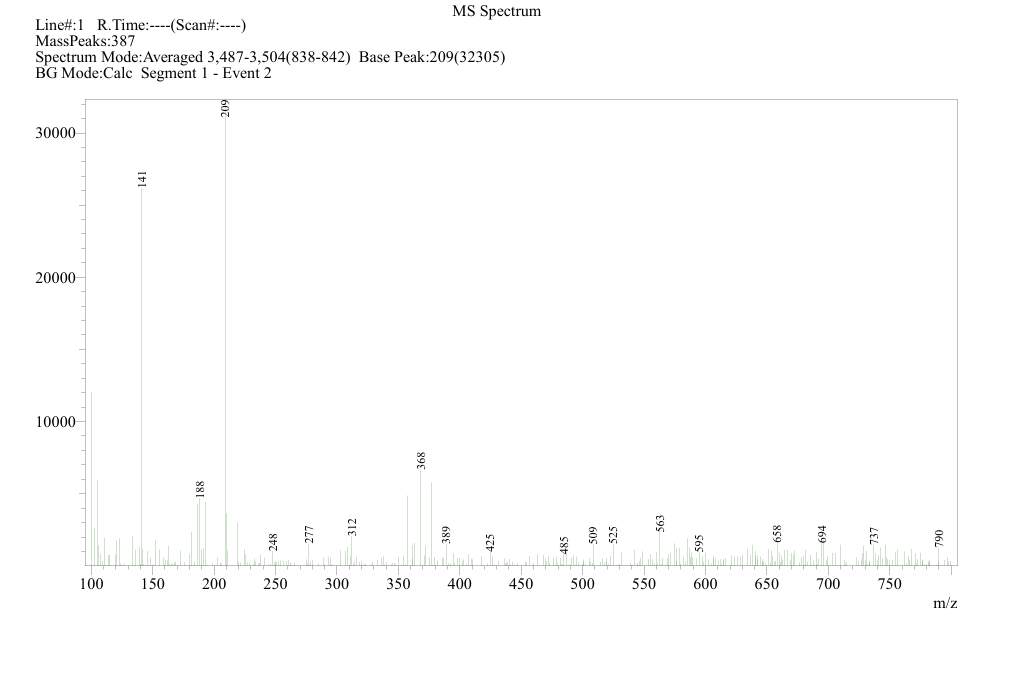


Chromatogram of Red seaweed Sulfated polysaccharide obtained by hot extraction

Supplement: Multimedia component 1 [file mmc1.docx]
